# Repeated Hepatic Dearterialization for Unresectable Liver Metastases From Gastric Cancer: Review of Five Cases

**DOI:** 10.1155/1995/96946

**Published:** 1995

**Authors:** Takeo Kimoto, Naofumi Nagasue, Hitoshi Kohno, Yu-Chung Chang, Hiroyuki Taniura, Akira Yamanoi, Masaaki Uchida, Yoshinari Takemoto, Yoshinari Makino, Teruhisa Nakamura

**Affiliations:** From Second Department of Surgery Shimane Medical University Izumo 693 Japan

## Abstract

A novel method of repeated hepatic dearterialization was evaluated in five patients with multiple metastases from gastric cancer in both hepatic lobes. After gastrectomy with extensive lymph node dissection (R2/3), all patients underwent implantation of a vascular occluder around the hepatic artery. Cannulation of the hepatic artery was added for later chemotherapy. The hepatic artery was occluded repeatedly for 1 hour twice daily in combination with intrahepatic infusion of anticancer drugs for as long as possible. Three of five patients demonstrated marked tumour regression with unexpectedly long survival (16 months in two patients and one still alive at 15 months). Carcinoembryonic antigen (CEA) levels decreased to almost normal in four patients who had initially high levels. The present experiences seems to indicate that long survival can be hoped for in patients with advanced gastric cancer with unresectable liver metastases.


*HPB Surgery*, 1995, Vol. 8, pp. 175–180
Reprints available directly from the publisher
Photocopying permitted by license only

© 1995 Harwood Academic Publishers GmbH
Printed in Malaysia

### Repeated Hepatic Dearterialization for Unresectable Liver Metastases from Gastric Cancer: Review of Five Cases

TAKEO KIMOTO, NAOFUMI NAGASUE, HITOSHI KOHNO, YU-CHUNG CHANG, HIROYUKI TANIURA, AKIRA YAMANOI, MASAAKI UCHIDA, YOSHINARI TAKEMOTO, YOSHINARI MAKINO and TERUHISA NAKAMURA

From Second Department of Surgery, Shimane Medical University, Izumo 693, Japan

A novel method of repeated hepatic dearterialization was evaluated in five patients with multiple metastases from gastric cancer in both hepatic lobes. After gastrectomy with extensive lymph node dissection (R2/3), all patients underwent implantation of a vascular occluder around the hepatic artery. Cannulation of the hepatic artery was added for later chemotherapy. The hepatic artery was occluded repeatedly for 1 hour twice daily in combination with intrahepatic infusion of anticancer drugs for as long as possible. Three of five patients demonstrated marked tumour regression with unexpectedly long survival (16 months in two patients and one still alive at 15 months). Carcinoembryonic antigen (CEA) levels decreased to almost normal in four patients who had initially high levels. The present experiences seems to indicate that long survival can be hoped for in patients with advanced gastric cancer with unresectable liver metastases.

KEY WORDS: Gastric cancer liver metastasis dearterialization hepatic artery

### INTRODUCTION

The prognosis of gastric cancer has become much better in Japan than in Western countries, mainly because of the increasing proportion of "curative resections" because of earlier detection of the disease[1]. However, advanced gastric cancers with peritoneal dissemination, hepatic metastases or widespread nodal involvement are also not infrequent in our country and the prognosis is still dismal for such patients. In advanced gastric cancer, liver metastases is one of the most ominous signs for prognosis[2] and the most common site of involvement[3]. Natural history of gastric cancer with the extensive liver metastases is less than two months[4]. This report describes our experience of five patients with gastric cancer who had unresectable multiple liver metastases, treated by a novel method of repeated hepatic dearterialization after aggressive resection of the original lesions.

*Address for correspondence:* Mr. T. Kimoto, Second Department of Surgery, Shimane Medical University, Izumo 693, Japan

### MATERIAL AND METHODS

### Patients

Between April, 1989 and February, 1992, five patients, four men and one woman, were treated by repeated hepatic arterial occlusion with intra-arterial infusion of anticancer drugs. The ages ranged between 52 and 73 years old. All patients had multiple liver metastases from gastric cancer without peritoneal dissemination. The liver tumours were found synchronously in four patients and metachronously in one (Table 1).

### Operative technique

Gastrectomy with R2-R3 lymph node dissection was performed in all patients. Four patients underwent partial gastrectomy and the other total gastrectomy. Hepatic bisegmentectomy (VI and VII) was performed in one patient, with multiple liver recurrence after six months. Implantation of vascular occluder was performed simultaneously in four patients, and metachronously in one patient. After dissection of all liver attachments, the

**Table 1** Summary of five cases

| *Case* | *Sex* | *Age* | *TNM Classification* | *Total doses of infusion chemotherapy* | *Survival time (Cause of death)* | *Reduction of hepatic tumours* |
|---|---|---|---|---|---|---|
| 1 | M | 59 | T3N3M1 | MMC 48mg, 5-FU 7000 mg | 16 months (Extrahepatic cancers) | 77% |
| 2 | F | 52 | T3N2M1 | MMC 46mg, 5-FU 19750 mg | 16 months (Extrahepatic Cancers) | 93% |
| 3 | M | 72 | T3N0M1 | MMC 44 mg, 5-FU 1000 mg ADR 45 mg | 4 months (Liver failure) | 35% |
| 4 | M | 73 | T2N0M1 | MMC 4 mg Epirubicin 200 mg | 2 months (Liver failure) | 17% |
| 5 | M | 66 | T3N3M1 | MMC 56.8 mg Epirubicin 284 mg | 15 months (alive) | 89% |

vascular occluder was placed round the common hepatic artery, and it was fixed to and wrapped with a sheath of expanded polytetrafluoroethylene (PTFE). The PTFE sheath was sutured encircling the artery and the balloon occluder together. The end of the catheter from the balloon was pulled out through the abdominal wall. Injection of 1 to 2 ml of saline could occlude the hepatic artery by compression with the balloon. The necessary volume for complete occlusion was confirmed at operation. The cannula for chemotherapy was inserted into the gastroduodenal artery. The right gastric artery was routinely ligated at its origin. (Fig. 1)

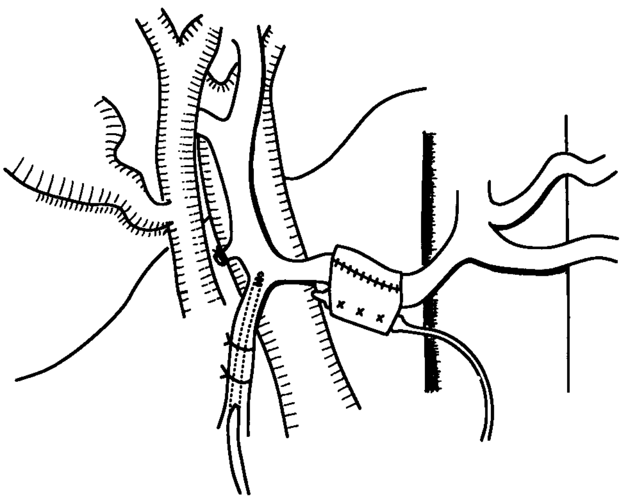

**Figure 1** The vascular balloon occluder is placed beside the common hepatic artery. They are wrapped together using expanded PTFE vascular sheath. Injection of 1 to 2 ml. of saline can compress the hepatic artery. The right gastric artery is routinely ligated.

*Postoperative treatment*

The occlusion was started from 5 to 8 days after operation. It was performed for 1 hour twice daily and repeated for as long as possible. Three patients were instructed how to occlude the hepatic artery and continued this therapy by themselves at home. Intra-arterial infusion of 5-Fluorouracil (5-FU), Mitomycin (MMC), Adriamycin (ADR) or Epirubicin were started from 8 to 11 days after operation and intermittently repeated for long as possible. Three patients were prescribed Carmofur (HCFU), 300 mg/day, orally after discharge from hospital. The effectiveness of the treatment was evaluated by carcinoembryonic antigen (CEA) and computed tomography (CT).

### CASE REPORTS

*Case 1*

This 59-year-old man had multiple liver metastases from gastric cancer. Both lobes of the liver were involved with tumours. The primary gastric cancer was located in the antrum and partially invaded the duodenum. There was no peritoneal dissemination but involvement of regional and other intra-abdominal lymph nodes such as para-aortic, hepatoduodenal and retropancreatic. He underwent partial gastrectomy, and extensive dissection of these lymph nodes. A vascular occluder was placed round the common hepatic artery and the cannula inserted. Hepatic arterial occlusion was started 8 days after operation for one hour twice a day and repeated for nine months. The occlusion time was reduced to once a day for a further three months. Intra-arterial infusion of MMC in a dose of 24 mg and 5-FU, 500 mg was added on the 8th postoperative day (POD). At 22 POD, the same doses were administered. From 28 POD for the next 12 days, 5-FU in a dose of 250 mg/day was infused daily. HCFU, 300

mg/day was given orally for 10 months after his discharge from hospital. Four months after the start of treatment, all tumours had became cystic lesions and showed 77 per cent reduction on CT scan. Serial CEA level became normal from 105 ng/ml at 3 POD to 0.6 ng/ml four months later. The occlusion therapy was continued by the patient at home. However, he died of extrahepatic recurrence in lymph nodes 16 months after the gastrectomy.

*Case 2*

This 52-year-old woman had numerous scattered metastases in both hepatic lobes. The primary gastric tumour was in the cardia. She underwent total gastrectomy with lymph node dissection including the left gastric, splenic, celiac and common hepatic arterial regions. Microscopic metastases were found in the regional and splenic nodes. Implantation of the vascular occluder and the cannula was also performed. The occlusion therapy for 1 hour twice a day was started at 5 POD and continued for 1 year. MMC, 10 mg and 5-FU, 250 mg were administered intra-arterially at 11 POD and the infusion of 5-FU in a dos of 250mg/day was repeated for 19 consecutive days. She repeatedly had these cytotoxic drugs, total doses of which were 46 mg for MMC and 19750 mg for 5-FU as an out-patient. Oral administration of HCFU 300mg/day was also added from 3 months to 5 months after operation. Five months after the therapy started the multiple metastatic lesions in the entire liver almost disappeared and all the residual lesions were cystic (Fig. 2) The value of CEA decreased from 75.4 ng/ml to 4.6 ng/ml by the same time. She lived a normal life and continued the occlusion therapy at home for a year. She however died owing to intra-abdominal lymph node and bone metastases though the hepatic tumours remained well controlled.

*Case 3*

This 72-year-old man underwent partial gastrectomy with R2 lymph node dissection. Lymph node metastases were not found histologically. For a massive metastasis in the right hepatic lobe, hepatic bisegmentectomy (segment VI and VII) was performed. However, 6 months after operation, multiple recurrences were found in the remnant liver. Implantation of a vascular occluder and cannulation were performed. The occlusion therapy was repeated from 7 POD but it was ceased at 24 POD due to liver dysfunction. The hyperbilirubinemia required plasmapheresis at 41 POD and 63 POD. The occlusion therapy was restarted at 51 POD with an expectation that regression of tumours by the treatment would allow the liver function to recover. However, the patient died of systemic bleeding due to liver failure. Ironically, autopsy revealed total necrosis of all the hepatic tumours.

This case urged us to reconsider the indication and timing of the therapy.

*Case 4*

This 73-year-old patient underwent partial gastrectomy with R2 lymph node dissection. Microscopic invasion of the resected nodes was not seen. The liver was involved with numerous massive tumours in the both lobes. After hepatic dearterialization, the entire surface of liver was covered with a polyethylene sheath to prevent collateral development. The occlusion therapy was started from 8 POD in spite of a high total bilirubin 132 µm/l. The patient tolerated the procedure but CT scan revealed a left subphrenic abscess after 3 weeks therapy, which was drained the next day. However, he died due to liver failure triggered by the abscess.

*Case 5*

This 66-year-old man had undergone partial gastrectomy with R3 lymph node dissection. Regional and hepatoduodenal lymph node metastases were found histologically. A massive tumour occupied the right hepatic lobe and the numerous scattered metastases were seen in both lobes. The occlusion therapy was started at 8 POD. Intra-arterial infusions of MMC in a dose of 2 mg/day everyday and Epirubicin, 10 mg, twice a week were repeated for a month. Additional administrations were repeated thereafter. The ischemic therapy was also repeated for 4 months but then ceased due to rupture of the balloon occluder. Three months after the therapy, the massive metastasis in the right lobe changed into a calcified lesion and other small tumours almost disappeared. The CEA level also decreased from 302 ng/ml at 13 POD to 10.7 ng/ml at 4 months. He is alive at 15 months after the initiation of the current therapy although minute lung metastases are present.

### DISCUSSION

The concept of transient hepatic dearterialization for liver tumours was first proposed in 1976[5], after a number of strategies such as hepatic artery ligation and hepatic dearterialization, in combination with or without anticancer drug infusion[6–8]. However, the result of conventional hepatic dearterialization was not satisfactory except for metastatic carcinoid disease[9] in terms of survival and complication rates as compared with intra-arterial infusion of 5-Fluorouracil alone[10]. This new method of

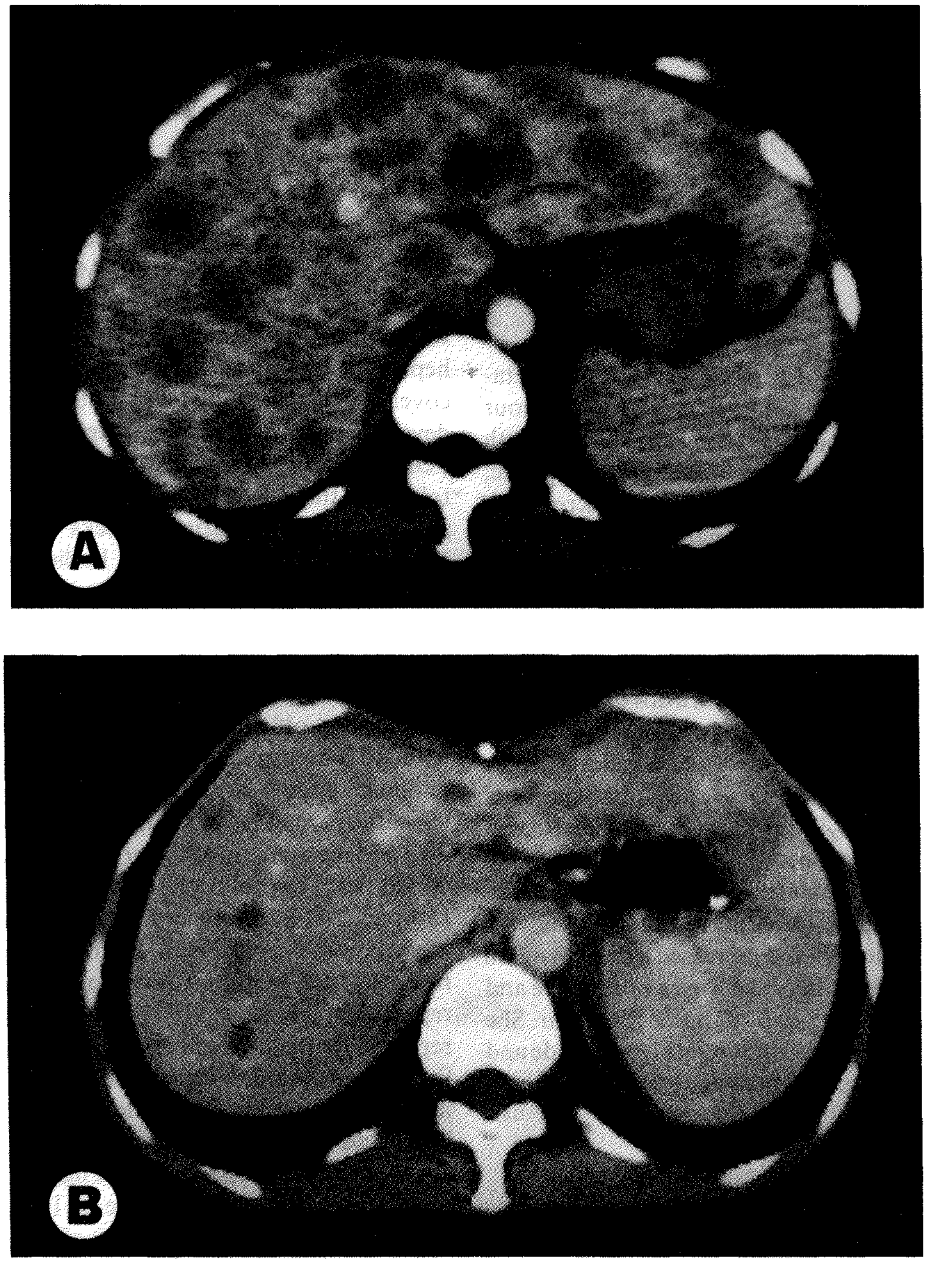

**Figure 2** CT scans in case 2. A before operation. B 5 months after the occlusion therapy.

repeated short-term occlusion of the hepatic artery was developed by Bengmark *et al.*[11–13] following studies showing that it can prevent hepatic arterial collaterals[14]. The possible implication of oxygen free radicals in this therapy was suggested by the same group[15]. Only two out of thirteen patients developed collaterals during the repeated temporary dearterialization and that at least six patients demonstrated the antitumour effect of this therapy[13].

We have also used this method for unresectable primary and secondary liver since 1988 and now present five cases of multiple liver metastases from gastric cancer. In all the five cases, tumour regression was obtained, among which three demonstrated more than 70% tumour reduction. The CEA levels also became normal or almost normal during the therapy in four patients. The remaining patient had a low CEA value at the start of the occlusion therapy (Fig.3). The density of tumours became lower by

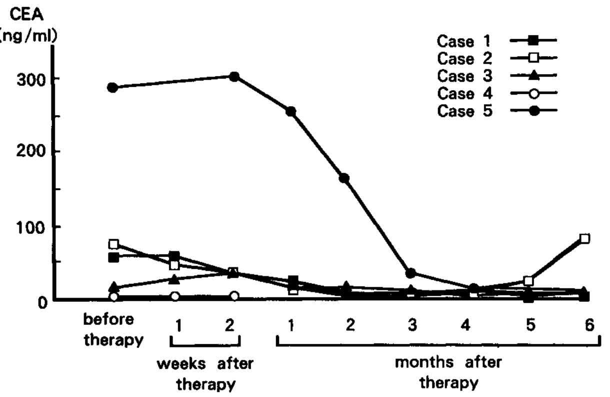

**Figure 3** Serial CEA changes for 6 months after start of the therapy in all cases.

CT examination following the therapy in four of five patients, which could indicate necrosis of the tumours. One patient exhibited massive tumour calcification as reported by other authors[11,13]. Three of five patients lived for 15 to 16 months, which had not been expected on admission. However, two other patients did not survive because of liver failure triggered by a subphrenic abscess or excessive ischemic therapy. These results may indicate that this therapy could be promising for treating multiple liver metastases from gastric cancer, provided the ability of the liver to tolerate the ischemic insult can be properly evaluated in each individual. Although this remains to be elucidated, the two patients with treatment failure in this study seem to show that in patients with too little liver parenchyma this treatment is contra-indicated.

In these five cases, intra-arterial chemotherapy with such drugs as MMC, 5-FU, ADR and Epirubicin were combined with the ischemic therapy. For liver metastases from gastric cancer, however, there is no study clearly showing that regional chemotherapy with these drugs alone can exert such a dramatic effect as seen in our patients. To clarify whether the ischemic therapy had an effect additional to regional chemotherapy, a randomized trial is needed.

The propriety of extended resectional surgery for the palliation of advanced gastric cancer is controversial. Some authors warned that aggressive procedures for palliative resection should be avoided because of their high operative mortality rate[16,17]. However, recent advances in surgical technique and postoperative care have markedly reduced the postoperative mortality in advanced gastric cancer[1,18]. Moreover, there is little doubt that resection of the tumour provides a better prognosis than bypass or intubation[19-21]. The five patients presented here underwent extended resections including nodal involvement, even in the presence of multiple liver metastases. Although all three patients who survived for a long time finally suffered from distant lymph node or lung metastases, the liver tumours remained well controlled. They had a good performance status with the repeated hepatic dearterialization therapy at home. These results seem to indicate that this therapy has some use in the treatment of unresectable liver metastases from gastric cancer.

### REFERENCES

1. Nagata, T., Ikeda, M., Nakayama, F. (1983) Changing state of gastric cancer in Japan. Histologic perspective of the past 76 years. *Am. J. Surg.*, **145**, 226–33.
2. Korenaga, D., Tsujitani, S., Haraguchi, M. *et al.* (1988) Long-term survival in Japanese patients with far advanced carcinoma of the stomach. *World J. Surg.*, **12**, 236–40.
3. Dupont, J. B., Lee J. R., Burton, G. R., Cohn, I. (1969) Adenocarcinoma of the stomach. Review of 1497 cases. *Cancer*, **41**, 941–7.
4. Bengmark, S., Hafstrom, L. (1969). The natural history of primary and secondary malignant tumors of the liver II. *Digestion*, **2**, 179–86.
5. El-Domeiri. (1976) A method of intermittent occlusion and chemotherapy infusion of the hepatic artery. *Surg. Gynecol. Obstet.*, **143**, 107–109.
6. Almerjo, O., Bengmark. S., Rudenstam, C. M. *et al.* (1972). Evaluation of hepatic dearterialization in primary and secondary cancer of the liver. *Am. J. Surg.*, **124**, 5–9.
7. Almerjo, O., Bengmark, S., Hafstrom, L., Leissner, K. H. (1976) Result of liver dearteriarization combined with regional infusion of 5-Fluorouracil for liver cancer. *Acta. Chir. Scand.*, **142**, 131–38.
8. Nagasue, N., Inokuchi, K., Kobayashi, M. *et al.* (1976) Hepatic dearterialization for nonresectable primary and secondary tumours of the liver. *Cancer*, **38**, 2593–603.
9. Bengmark, S., Ericsson, M., Lunderquist, A. *et al.* (1982) Temporary liver dearterialization in patients with metastatic carcinoid diseases. *World J. Surg.*, **6**, 46–53.
10. Ekberg, H., Tranberg, K. G., Lundstedt, C. *et al.* (1986) Determinants of survival after intra-arterial infusion of 5-Fluorouracil for liver metastases from colorectal cancer: A multivariate analysis. *J. Surg. Oncol.*, **31**, 246–254.
11. Bengmark, S., Jeppsson, B., Lunderquist, A. *et al.* (1988) Tumour calcification following repeated hepatic de-arterializa-tion in patients: a preliminary communication. *Br. J. Surg.*, **75**, 525–6.
12. Bengmark, S., Jeppsson, B. (1989) Status of ischemic therapy for hepatic tumors. *Surgical Clinics of North America*, **69**, 411–18.
13. Persson, B. G., Jeppsson, B., Ekberg, H. *et al.* (1990) Repeated dearterialization of hepatic tumors with an implantable occluder. *Cancer*, **66**, 1139–46.
14. Persson, B. G., Jeppsson, B., Andersson, L. *et al.* (1987) The prevention of arterial collaterals after repeated temporary blockade of the hepatic artery in pigs. *World J. Surg.*, **11**, 672–77.
15. Puntis, M. C. A., Persson, B., Jeppsson, B. *et al.* (1987) Free radical production in the ischemic rat liver. *Surg. Res. Comm.*, **1**, 17–20.
16. Gilbertson, V. A. (1969) Result of treatment of stomach cancer: An appraisal of more extensive surgery and a report of 1983 cases. *Cancer*, **23**, 1305–8.

17. ReMine, W. H. (1979) Palliative operations for incurable gastric cancer. *World J. Surg.*, **3**, 721–29.
18. Lawrence, S. B. (1983) Adenocarcinoma of the stomach: Current results of treatment. *Cancer*, **51**, 743–45.
19. Koga, S., Kawaguchi, H., Kishimoto, H. *et al.* (1980) Therapeutic significance of noncurative gastrectomy for gastric cancer with liver metastasis. *Am. J. Surg.*, **140**, 356–59.
20. Meijer, S., De Bakker, O. J. G. B., Hoitsma, H. F. W. (1983) Palliative resection in gastric cancer. *J. Surg. Oncol.*, **23**, 77–80.
21. Haugstvedt, T., Viste, A., Eide, G. E. *et al.* (1989) The survival benefit of resection in patients with advanced stomach cancer: The Norwegian multicenter experience. *World J. Surg.*, **13**, 617–22.

### COMMENTRY

This is an interesting report on 5 patients with advanced gastric cancer that has proved to be very resistant to almost any kind of treatment. Most surgeons would hesitate to perform such aggressive treatment because of the very poor prognosis indeed. Although an occasional patient may have a long survival even without treatment it is hard to imagine that they would survive for such long periods as was seen in three of the patients. I find it striking that they all had a dramatic response, radiological as well as biochemical. To my knowledge chemotherapy alone has not been able to show the same response, the addition of repeated dearterializations must have been responsible. Previous attempts to use ischemic therapy have been disappointing. Considerable palliation seems to have been offered to these five patients with tolerable side effects; three of them managed to perform the dearterializations at home.

One can argue that the report comprises only five patients and , of course, the chance can never be ruled out. However, based on these favourable responses and survival a randomised trial would seem appropriate.

Both hepatic artery ligation and the more extensive permanent dearterilization fail to achieve a sustained tumour growth delay because of a speedy development of collaterals. Intermittent but repeated dearterialization seems to be able to offer a longer growth delay in part owing to a prevention of collaterals. We have previously used dearterialization for 1 hour/day. However, based on recent results (in a rat liver-tumour model) 2 hours/day seems to be the optimal period in retarding liver tumour growth[1] and has led us to change our protocol.

1. Repeated dearterialization of an experimental liver tumour: short-and long-term results.

Li Qing Wang , Bo G Persson and Stig Bengmark. J Surg Res; In Press

Bo G Persson
Lund, Sweden

